# Latent Factor Modeling of Four Schizotypy Dimensions with Theory of Mind and Empathy

**DOI:** 10.1371/journal.pone.0113853

**Published:** 2014-11-21

**Authors:** Jeffrey S. Bedwell, Michael T. Compton, Florian G. Jentsch, Andrew E. Deptula, Sandra M. Goulding, Erin B. Tone

**Affiliations:** 1 University of Central Florida, Department of Psychology, Orlando, Florida, United States of America; 2 Department of Psychiatry, Hofstra North Shore–LIJ School of Medicine at Hofstra University, Hempstead, New York, United States of America; 3 Graduate School of Arts and Sciences of Emory University, Department of Psychology, Atlanta, Georgia, United States of America; 4 Georgia State University, Department of Psychology, Atlanta, Georgia, United States of America; University of Rochester, United States of America

## Abstract

Preliminary evidence suggests that theory of mind and empathy relate differentially to factors of schizotypy. The current study assessed 686 undergraduate students and used structural equation modeling to examine links between a four-factor model of schizotypy with performance on measures of theory of mind (Reading the Mind in the Eyes Test [MIE]) and empathy (Interpersonal Reactivity Index [IRI]). Schizotypy was assessed using three self-report measures which were simultaneously entered into the model. Results revealed that the Negative factor of schizotypy showed a negative relationship with the Empathy factor, which was primarily driven by the Empathic Concern subscale of the IRI and the No Close Friends and Constricted Affect subscales of the Schizotypal Personality Questionnaire. These findings are consistent with a growing body of literature suggesting a relatively specific relationship between negative schizotypy and empathy, and are consistent with several previous studies that found no relationship between MIE performance and schizotypy.

## Introduction

*Schizotypy* refers to a set of personality traits that vary in the general population along a continuum that ranges from no formal diagnosis and minimal impairment, to schizotypal, paranoid, and avoidant personality disorders, and to psychotic disorders like schizophrenia [1]–[3]. Conditions along this dimension are thought to share genetic links with schizophrenia [4]–[6]. Therefore, examinations of psychological features of subclinical schizotypy offer insight into schizophrenia that is untainted by the effects of confounding variables such as chronic neuroleptic use, severe active symptomatology, and hospitalizations [7]–[8].

Many individuals with schizophrenia have pervasive social-cognitive impairments that include deficits on measures of theory of mind and empathy [9]–[11]. In nonpsychiatric samples, reduced performance on theory of mind tasks is primarily related to the positive features of schizotypy (e.g., magical ideation and unusual perceptual experiences) [12]–[18]. In contrast, reduced self-reported ratings of empathy have been reported to show the strongest relationships with negative features of schizotypy (e.g., social anhedonia, social anxiety, and constricted affect) [15], [19], [20], and the two studies which examined a disorganized factor also found a significant negative relationship between disorganized schizotypy and empathy [15], [20].

Commonly used measures of schizotypy assess different schizotypal features. For example, the Chapman Scales of Psychosis Proneness [21], [22], assess social anhedonia but not social anxiety, while the Schizotypal Personality Questionnaire [23] includes social anxiety along with a broader range of features. Because studies exploring the construct's associations with social cognition have assessed schizotypy using a variety of measures, each of which taps different facets, cross-study comparisons are difficult to make.

Surprisingly few studies have examined relationships among the social cognitive domains of empathy and theory of mind and multiple domains of schizotypy; with research typically only reporting relationships with overall schizotypy or its broad domains (e.g., positive vs. negative aspects). As a step toward addressing these knowledge gaps, the present study assessed schizotypy—measured using three self-report scales—in a large sample of undergraduate students. We used structural equation modeling to determine how four latent factors of schizotypy related to latent factors of theory of mind and empathy.

Based on the existing research, we hypothesized that structural equation modeling would reveal a significant negative relationship between factors of positive schizotypy (i.e., Cognitive-Perceptual and Paranoid) and theory of mind, while simultaneously showing a negative relationship between factors of negative and disorganized schizotypy and empathy.

## Materials and Methods

### Ethics Statement

The study was approved by the Institutional Review Board (IRB) of Georgia State University. The IRB waived the requirement to document consent. However, participants read a consent statement online, prior to starting study, and were told that they were providing implicit consent by proceeding onto the rest of the study if they chose to. The investigation was conducted according to the principles expressed in the Declaration of Helsinki.

### Setting and Sample

Individuals aged ≥18 years who were enrolled in introductory psychology courses at a public, urban university were invited to participate. Following informed consent, participants completed all measures online in a randomized order. From the 793 participants who completed the measures of interest, we excluded 81 participants who completed the full assessment at a pace that was faster than that of 90% of the group (<26 min.; mean  = 45.38 min.; SD  = 22.34) in order to reduce the possibility of random responding and/or poor attention to item content. We also excluded 26 participants who did not complete one of the study measures (SAS – described below). This resulted in a final sample of 686 participants whose data were included in analyses.

### Measures

The 74-item *Schizotypal Personality Questionnaire* (SPQ) [23] addresses all nine *Diagnostic and Statistical Manual of Mental Disorders, Fourth Edition, Text Revision* (DSM-IV-TR; American Psychiatric Association, 2000) diagnostic criteria for schizotypal personality disorder using a true/false selection. Items are grouped into nine subscales: Ideas of Reference, Magical Thinking, Unusual Perceptual Experiences, Suspiciousness, Social Anxiety, No Close Friends, Constricted Affect, Eccentric Behavior, and Odd Speech. Past studies support the psychometric properties of the SPQ, with internal consistency of *r* = .90, test-retest reliability of *r* = .82, and convergent and criterion validity ranging from *r* = .59 to.81 [23].

The 35-item *Perceptual Aberration Scale* (PAS) [22] is a true/false measure designed to operationalize body-image distortions and perceptual anomalies. Extensive past research demonstrates that the PAS is a reliable (internal consistency, *r* = .79–.89) and valid indicator of schizotypal traits in both clinical and non-clinical populations [24], [25]. The 40-item *Revised Social Anhedonia Scale* (SAS) [21], [26] is a true/false measure assessing deficits in the ability to experience pleasure from interpersonal interactions. The SAS has been used extensively in clinical and non-clinical populations, has shown good reliability (internal consistency, *r* = .84), appears to be relatively independent of other measures of psychosis-proneness (including the PAS), and identifies individuals exhibiting significant social maladjustment [27], [28].

The 28-item *Interpersonal Reactivity Index* (IRI) [29] is a multi-dimensional self-report assessment of empathy consisting of four subscales: Perspective-Taking, Fantasy, Empathic Concern, and Personal Distress. The Perspective-Taking subscale measures a tendency to adopt others' points of view; the Fantasy subscale assesses one's likelihood of identifying with fictional characters; and the Empathic Concern subscale measures feelings of concern, warmth, and sympathy toward others [29], [30]. The Personal Distress subscale was omitted from the present study as it does not assess empathy. Participants rate how well each item describes them using a 5-point Likert scale (0 =  does not describe me well, to 4 =  describes me very well). Ratings are summed to yield domain scores, with higher scores indicating greater levels of empathy. Reliability and validity estimates for the measure's subscales are adequate to good in a range of culturally varied samples (e.g., [31]), with estimated internal consistency of *r* = .84 [42].

The *Reading the Mind in the Eyes Test, Revised* (MIE) [32] is a 36-item measure of theory of mind that requires individuals to characterize mental states based on nonverbal cues conveyed by the eyes. Participants view 36 photographed pairs of eyes and select which of four complex mental state descriptions (e.g., playful, comforting, irritated, bored) best describes the internal state depicted in each photo. The total score is the sum of the correct responses. Baron-Cohen and colleagues [32] demonstrated that the measure has acceptable construct validity.

### Sample Characteristics

The mean age of the 686 participants (77% female) was 21.22 (SD = 4.40; range 18 to 52). Slightly less than half (46.4%) self-identified their race as “White/Caucasian”, while 30.9% identified as “Black/African American”, 9.3% as “Asian American”, 6.1% as “Biracial/Multicultural”, 0.7% as “American Indian/Native American”, 0.3% as “Native Hawaiian/Pacific Islander”, and 6.3% as “Other”. Independent of these racial categories, 8.5% indicated an ethnicity of “Latino(a)/Hispanic”. Five participants (0.007%) reported that a biological relative had been diagnosed with schizophrenia.

### Data Analyses

Distributional properties of all variables were examined. Structural equation modeling was conducted using IBM SPSS Amos 21.0. During model specification, the error variance for second-level latent variables with only two indictors was set to 1. The following fit statistics were examined for each model and are presented in Table 1: chi-squared difference test, Steiger-Lind root-mean square error of approximation (RMSEA), Bentler's comparative fit index (CFI), Akaike information criteria (AIC), and the James et al. parsimonious normal fit index (PNFI). Based on commonly accepted fit values, for the RMSEA we used a cutoff of <0.10 as acceptable and <.05 as good. For the CFI we used a cutoff of >0.90 as acceptable and >0.95 as good. The AIC and PNFI do not have commonly accepted cutoff values in the field, but relatively smaller AIC values represent better fit, while relatively larger PNFI values represent better fit.

**Table 1:** Fit statistics for models examined.

Model Examined	Χ^2^ Diff.	RMSEA	CFI	AIC	PNFI
Schizotypy 3 factor model	493 ***	.125	.857	563	.536
Schizotypy 4 factor model (**selected**)	292 ***	.097	.920	368	.646
Social Cognition 1 factor model	223 **	.253	.575	253	.287
Social Cognition 2 factor model (**selected**)	10.54 *	.049	.987	43	.392
Model linking schizotypy to social cognition	487 ***	.080	.896	611	.658
Model linking negative schizotypy to empathy	140 ***	.097	.924	190	.620

*  = p<.05, **  = p<.01, ***  = p<.001.

Χ^2^ Diff.  =  chi-squared difference test; RMSEA  =  Steiger-Lind root-mean square error of approximation, CFI  =  Bentler's comparative fit index; AIC  =  Akaike information criteria; PNFI  =  James et al. parsimonious normal fit index.

**Figure 1:**
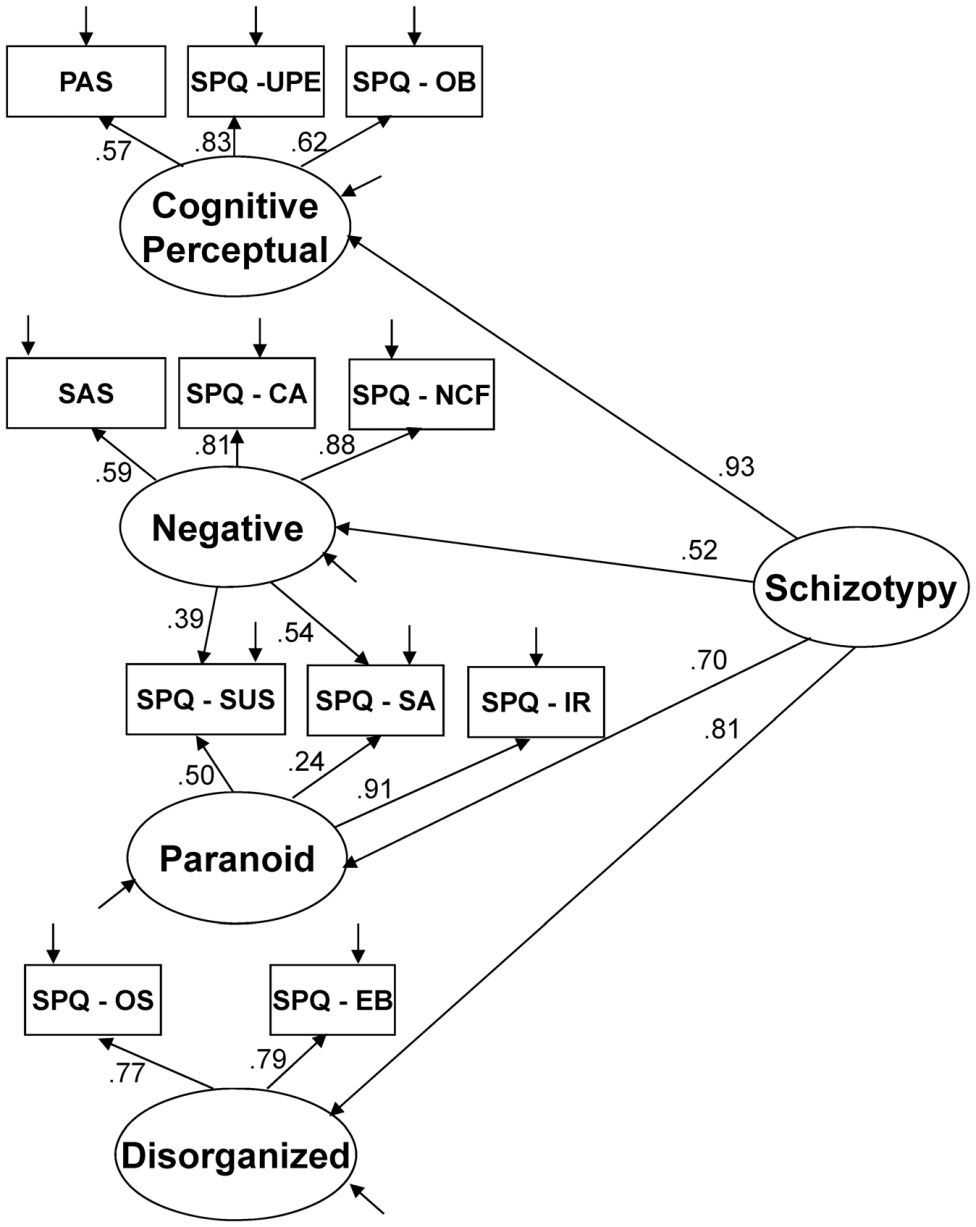
Structural equation model of the chosen four-factor model of schizotypy. PAS  =  Perceptual Aberration Scale; UPE  =  Unusual Perceptual Experiences; OB  =  Odd Beliefs; SAS  =  Revised Social Anhedona Scale; CA  =  Constricted Affect; NCF  =  No Close Friends: SA  =  Social Anxiety; SUS  =  Suspiciousness; IR  =  Ideas of Reference; OS  =  Odd Speech; EB  =  Eccentric Behavior.

Prior to including social cognition variables in the model, we first examined the fit of a schizotypy model. We started by examining the 4-factor model for the SPQ proposed by others [33], [34], particularly as our sample partially overlapped the sample used in one of these studies [33]. As we administered two additional measures of schizotypy (SAS and PAS) that were not included in these past factor analytic studies, we added these to the existing four-factor model using theoretical assumptions by placing the SAS score on the Negative factor, and PAS on the Cognitive-Perceptual factor. All connections between the SPQ subscales with the four factors were chosen based on the existing research on the four-factor SPQ model [33], [34]. Examination of resulting modification indices did not suggest any changes to these placements. This final four-factor schizotypy model, which is displayed in Figure 1, produced a fair fit (see Table 1), and all paths were significant at *p*<.001. An attempt to fit the same scales onto the traditional three-factor model of the SPQ [23] (and adding the SAS on the Interpersonal Factor and the PAS on the Cognitive-Perceptual Factor) produced a poorer fit across all indices than the four-factor model (see Table 1). As previous studies using the SPQ have relied on either three or four factor models e.g., [23], [33], [34], we did not explore additional potential models (e.g., two or five), in order to allow more direct comparisons of our findings with other existing studies using the SPQ. We therefore decided to use the four-factor model of schizotypy depicted in Figure 1 for subsequent analyses.

**Figure 2:**
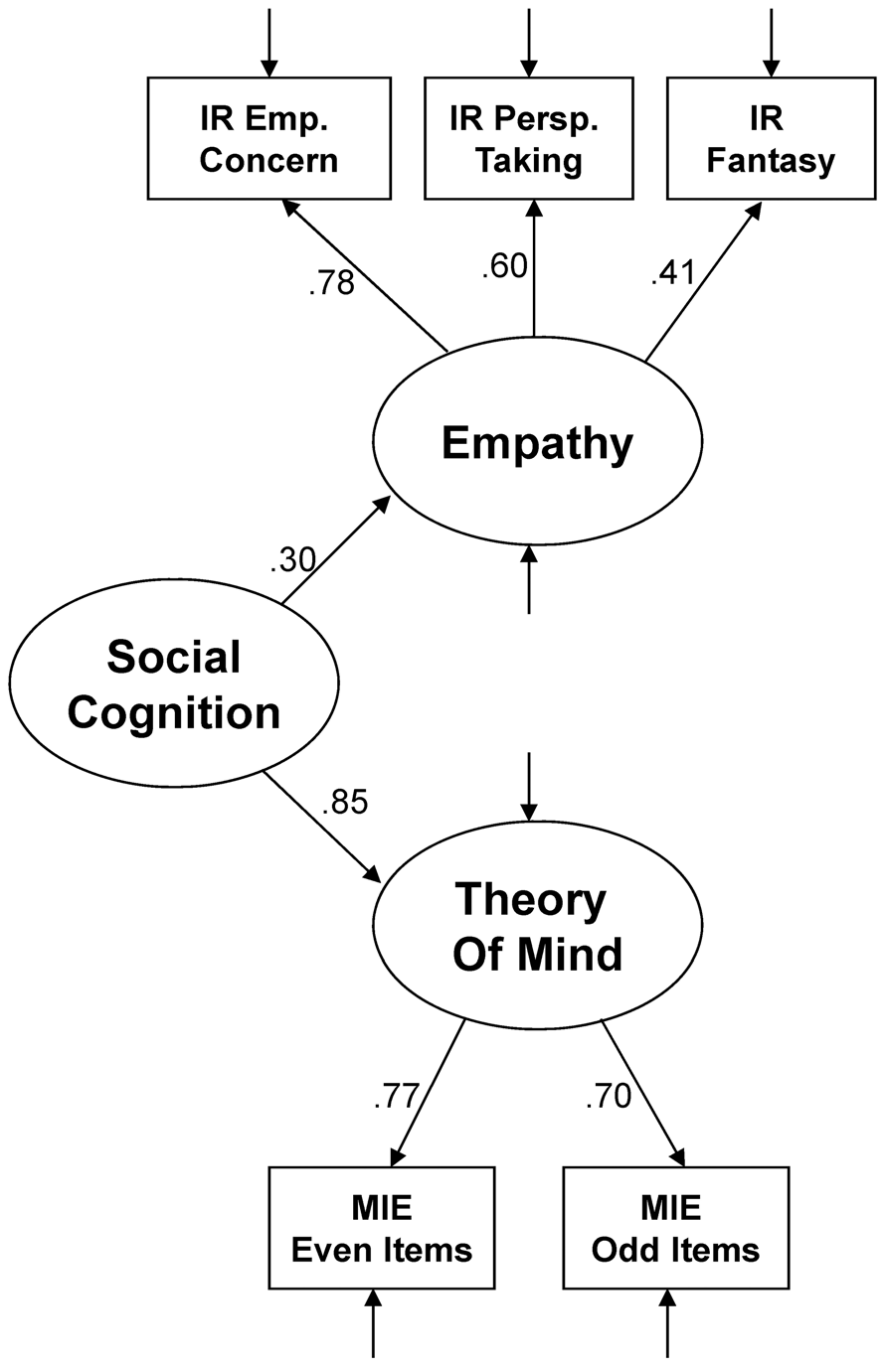
Structural equation model of the chosen model of social cognition. IR  =  Interpersonal Reactivity Index; Emp. Conc.  =  Empathic Concern; Persp. Taking  =  Perspective Taking; MIE  =  Reading the Mind in the Eyes Test, Revised.

Next we examined a theoretical latent model to account for the social cognition variables without including schizotypy – see Figure 2. Based on existing research indicating overlapping, yet distinct, elements in the theory and neural underpinnings of empathy and theory of mind [35], [36], we chose a model that contained an overall social cognition latent variable, which was linked to an empathy latent variable (with the three IRI subscales as indicators) and a theory of mind latent variable (with arbitrarily-defined even and odd items of the MIE as indicators, as at least two indicators are needed for a latent variable). This model produced a good fit (see Table 1), and all paths were significant at *p*<.001. This model had a notably better fit than that of an alternate model, which had a single latent social cognition variable directly connected to the three IRI subscales and the MIE indicators (see Table 1). We therefore decided to use the model of social cognition depicted in Figure 2.

**Figure 3:**
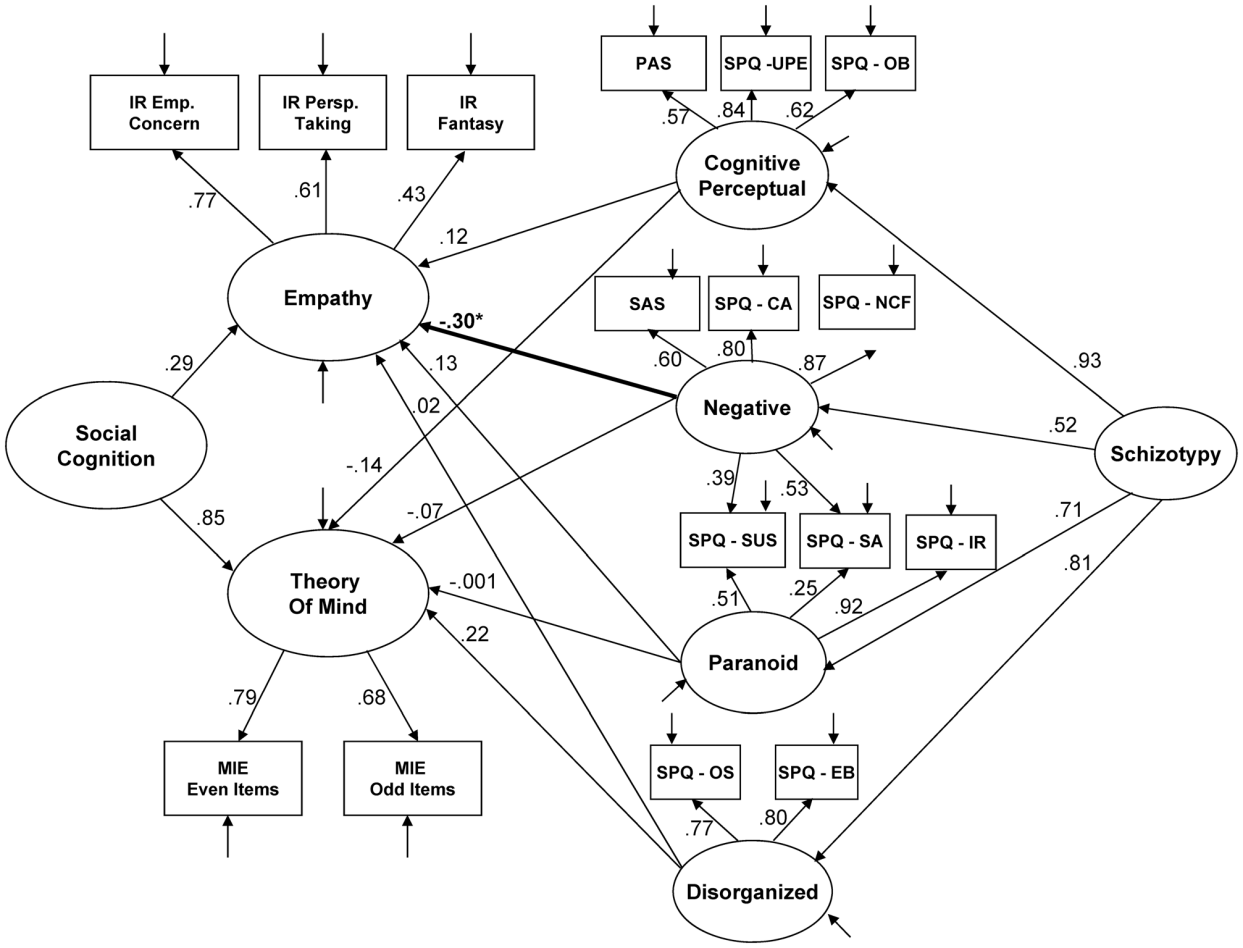
Full structural equation model linking social cognition to schizotypy. PAS  =  Perceptual Aberration Scale; UPE  =  Unusual Perceptual Experiences; OB  =  Odd Beliefs; SAS  =  Revised Social Anhedona Scale; CA  =  Constricted Affect; NCF  =  No Close Friends: SA  =  Social Anxiety; SUS  =  Suspiciousness; SPQ IR  =  Ideas of Reference; OS  =  Odd Speech; EB  =  Eccentric Behavior; IR  =  Interpersonal Reactivity Index; Emp. Conc.  =  Empathic Concern; Persp. Taking  =  Perspective Taking; MIE  =  Reading the Mind in the Eyes Test, Revised. * *p*<.05 for the second order links.

After identifying the optimal schizotypy (Figure 1) and social cognition (Figure 2) models, we then created a third model which connected the constructs (see Figure 3). As we were primarily interested in the relationships between particular factors of schizotypy and particular aspects of social cognition, the links in the model were created accordingly. Specifically, the four schizotypy factors were each linked with the two social cognition factors. This allowed us to directly examine the specific hypotheses of the study, which predicted that positive features of schizotypy (i.e., the Cognitive-Perceptual and Paranoid factors) would be negatively related to the Theory of Mind factor, and that the Negative and Disorganized factors of schizotypy would be negatively related to the Empathy factor. The overall model included all eight paths from the four second level latent variables of Schizotypy (i.e., Cognitive-Perceptual, Negative, Paranoid, and Disorganized) to the two second level latent variables of Social Cognition (i.e., Empathy and Theory of Mind). In addition, we retained the higher-order factors of Schizotypy and Social Cognition in this model, as we wanted to examine the relative specificity of the factor relationships in the presence of the higher-order factors.

The full model linking the social cognition factors with the schizotypy factors showed a fair fit (see Table 1 and Figure 3).

## Results

Descriptive statistics for all measures can be found in Table 2, and zero-order correlations are presented in Table 3.

**Table 2:** Descriptive Statistics (N = 686).

	Mean (SD)	Range
Social Anhedonia (SAS)	9.33 (6.37)	0 to 36
Perceptual Aberrations (PAS)	4.67 (4.94)	0 to 33
Unusual Perceptual Experiences (SPQ)	2.20 (1.98)	0 to 9
Magical Ideation (SPQ)	1.45 (1.66)	0 to 7
Ideas of Reference (SPQ)	3.72 (2.58)	0 to 9
Suspiciousness (SPQ)	2.66 (2.17)	0 to 8
Eccentric Behavior (SPQ)	1.87 (2.14)	0 to 7
Odd Speech (SPQ)	3.04 (2.44)	0 to 9
No Close Friends (SPQ)	2.24 (2.21)	0 to 9
Constricted Affect (SPQ)	1.72 (1.76)	0 to 8
Social Anxiety (SPQ)	3.07 (2.18)	0 to 8
Theory of Mind (MIE)	23.95 (4.30)	4 to 34
Empathic Concern (IRI)	19.67 (4.64)	2 to 28
Perspective Taking (IRI)	17.38 (4.66)	1 to 28
Fantasy (IRI)	16.99 (5.59)	0 to 28

SAS  =  Social Anhedonia Scale Revised; PAS  =  Perceptual Aberration Scale; SPQ  =  Schizotypal Personality Questionnaire; MIE  =  Reading the Mind in the Eyes Test; IRI  =  Interpersonal Reactivity Index.

**Table 3:** Zero-order Pearson Correlation Coefficients (N = 686).

	1	2	3	4	5	6	7	8	9	10	11	12	13	14
1. Social Anhedonia (SAS)														
2. Perceptual Aberrations (PAS)	.20***													
3. Unusual Perceptual Experiences (SPQ)	.10**	.44***												
4. Magical Ideation (SPQ)	.06	.35***	.55***											
5. Ideas of Reference (SPQ)	.08*	.35***	.55***	.39***										
6. Suspiciousness (SPQ)	.30***	.29***	.43***	.27***	.59***									
7. Eccentric Behavior (SPQ)	.19***	.40***	.51***	.38***	.37***	.36***								
8. Odd Speech (SPQ)	.23***	.41***	.48***	.28***	.39***	.45***	.59***							
9. No Close Friends (SPQ)	.58***	.25***	.25***	.13***	.24***	.49***	.35***	.42***						
10. Constricted Affect (SPQ)	.44***	.34***	.35***	.21***	.31***	.48***	.41***	.55***	.69***					
11. Social Anxiety (SPQ)	.23***	.26***	.32***	.28***	.39***	.44***	.31***	.45***	.55***	.56***				
12. Theory of Mind (MIE)	−.12**	−.13**	.03	.01	.003	−.02	.06	.03	.01	−.02	.04			
13. Empathic Concern (IRI)	−.26***	−.14***	.03	.04	.06	−.02	−.08*	−.03	−.16***	−10**	−.01	.14***		
14. Perspective Taking (IRI)	−.19***	−.05	.08*	.14***	.006	−.03	.06	−.01	−.10*	−.06	−.04	.14***	.48***	
15. Fantasy (IRI)	−.11**	.13***	.21***	.15***	.23***	.11**	.18***	.15***	.002	.04	.12**	.18***	.33***	.22***

* *p*<.05, ** *p*<.01, *** *p*<.001.

SAS  =  Social Anhedonia Scale Revised; PAS  =  Perceptual Aberration Scale; SPQ  =  Schizotypal Personality Questionnaire; MIE  =  Reading the Mind in the Eyes Test; IRI  =  Interpersonal Reactivity Index.

**Figure 4:**
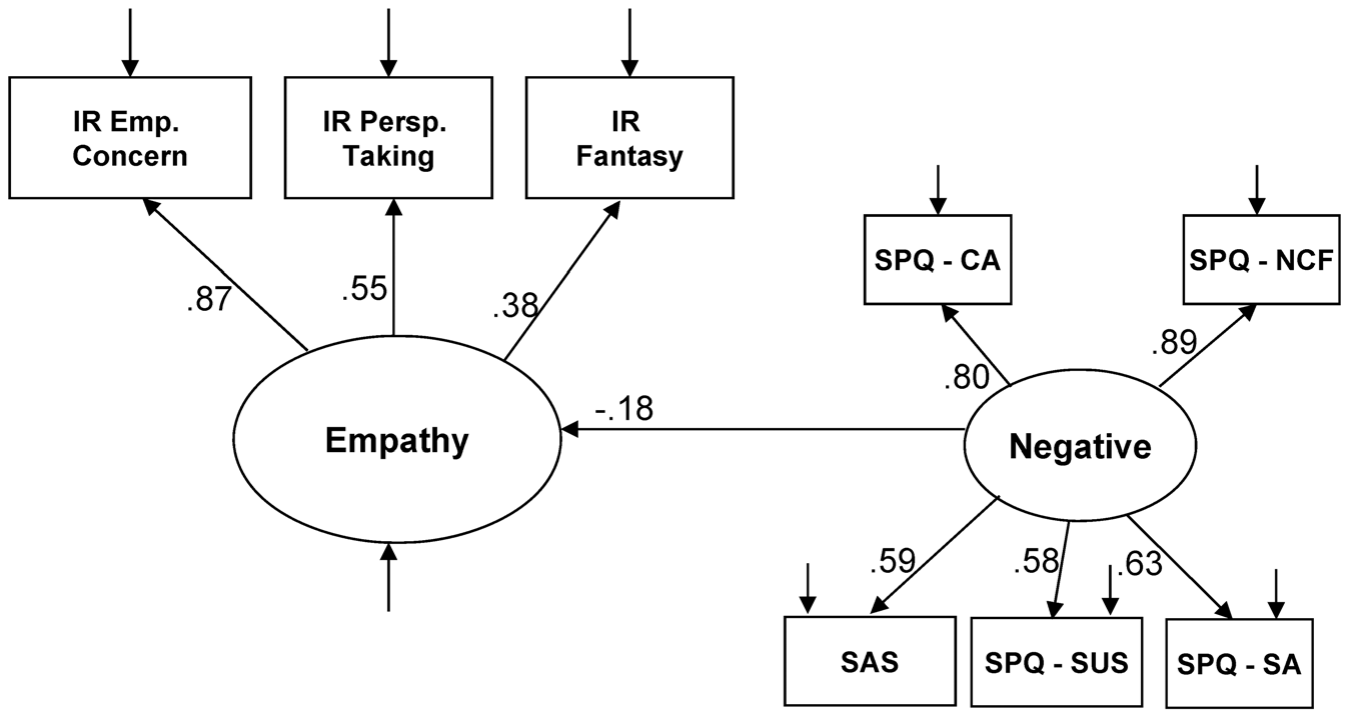
Structural equation model linking negative schizotypy to empathy. SAS  =  Revised Social Anhedona Scale; CA  =  Constricted Affect; NCF  =  No Close Friends: SA  =  Social Anxiety; SUS  =  Suspiciousness; IR  =  Interpersonal Reactivity Index; Emp. Conc.  =  Empathic Concern; Persp. Taking  =  Perspective Taking.

Examination of the eight paths linking the two social cognition latent variables to the four schizotypy latent variables revealed that only one path was statistically significant after a Bonferroni correction for the eight paths of interest (*p*<.006). The one significant path was a negative relationship (standardized coefficient  = −0.30, *p*<.001) between the Negative factor of schizotypy with Empathy. We further confirmed this finding by randomly dividing the sample into two subsets and running the same analysis, which revealed this same isolated finding within each subset, as was found in the entire sample. To further explore the subcomponents driving this relationship, we examined a fourth model which examined Empathy and the Negative factor of schizotypy in the absence of all other factors. The model showed a fair fit (see Table 1 and Figure 4). All paths in model were statistically significant (all *p*s <.001). Examination of the standardized coefficients in Figure 4 revealed that the Empathic Concern scale of the IRI (standardized coefficient  = .87) was the primary component of Empathy driving the relationship with the Negative factor of schizotypy, followed by the Perspective Taking (.55) and Fantasy (.38) subscales of the IRI. When examining the subscales of the Negative schizotypy factor, the No Close Friends (.89) subscale was the primary subscale driving the relationship with Empathy, followed by Constricted Affect (.80), Social Anxiety (.63), Social Anhedonia (.59), and Suspiciousness (.58).

## Discussion

As predicted, we found that the Negative factor of schizotypy showed a significant negative relationship with the Empathy factor. This is consistent with two previous studies using the same measure of empathy (IRI) [19], [20], as well a third study that used the Empathy Quotient measure [15]. Further examination revealed that the Empathic Concern subscale showed the strongest relationship with negative schizotypy, which was also found in two previous studies using the IRI [19], [20]. We also found that, of the five scales that loaded on the Negative factor, the No Close Friends and Constricted Affect subscales of the SPQ showed the strongest relationship with Empathy. It does not appear that previous research has explored the particular facets of negative features relating to empathy; thus, this finding remains preliminary. Inconsistent with our hypothesis, we did not find that the Disorganized factor of schizotypy showed a relationship with Empathy. It is possible that our use of structural equation modeling revealed a more specific relationship with the Negative factor after simultaneously accounting for the the other factors, which contrasts with the correlational approach used in the previous studies finding the relationship between disorganized features and empathy [15], [20].

Our findings did not support our hypothesis that facets of schizotypy would relate to the Theory of Mind factor, as we did not find any statistically significant relationship between any of the schizotypy factors with the Theory of Mind factor. This is inconsistent with findings of some earlier studies, which found a significant negative relationship between positive schizotypy in particular with various measures of theory of mind [12], [15]–[18]. However, it is consistent with three other studies that examined MIE performance in particular and found no relationship with positive or negative schizotypy [14], [37], [38].

However, the terms *theory of mind* and *empathy* label partially overlapping constructs as embodied representations of affect (i.e., emotional empathy) may contribute to, and be affected by, cognitive representations of the mental states of others [35]. In fact, a recent meta-analysis demonstrated that empathy and theory of mind performance elicit activity in an overlapping, but partially distinct, set of brain structures [36]. Furthermore, we inferred theory of mind from performance on the MIE test, which is a relatively objective measure of ability to infer the mental state of others based on a limited set of nonverbal cues. In contrast, empathy was self-reported (rather than demonstrated) in our study using subscales of the IRI, which assess not only how much the individual believes that he or she can engage in cognitive and emotional empathy, but also how frequently he or she experiences empathy in daily life. Effect sizes indicating the relatedness between all IRI subscales and MIE performance were small, although significant (*r* = .14 to.18), suggesting that these constructs were overlapping but distinct based on the measures included in the present study.

Several other methodological limitations should be noted when considering the applicability of findings from this study. First, although the sample was large, it was comprised of undergraduate university students; as such, findings may not generalize to individuals with schizotypy in the broader community. Second, it is difficult to determine the impact that response biases may have had on findings that involved examination of self-report measures. In addition, we investigated only one measure of theory of mind. Future studies should seek to examine the multidimensional aspects of theory of mind and its relationship with empathy. Finally, there is continued controversy over whether schizotypy is taxonic or fully dimensional. Nonetheless, a recent review supports the fully dimensional model that is used in the present study [39], and our primary interest was to examine the degree to which each dimensional facet of schizotypy showed differential relationships with measures of social cognition.

Despite these methodological limitations, the current study took a relatively novel approach of using structural equation modeling approach in a large sample to simultaneously examine how four dimensional factors of schizotypy relate to both empathy and theory of mind while accounting for overlapping variance among the constructs in a single model. Our results validate earlier findings of a robust relationship between negative schizotypy and empathy and suggest that theory of mind does not account for additional variance in a relationship with schizotypy; at least when theory of mind is inferred from performance on the MIE task. Results also support previous findings that the relationship with negative schizotypy is most robust with the Empathic Concern subscale of the IRI; a subjective report of feelings of concern, warmth, and sympathy toward others. Finally, we found that the No Close Friends and Constricted Affect subscales of the SPQ accounted for the most variance with the Negative factor in this relationship.

Overall, findings suggest that individuals who have few close personal relationships and a restricted range of affect also report feeling less warmth and sympathy toward others. Unfortunately the current study cannot infer the causal direction of this relationship, as it could theoretically occur in either direction. However, this relative lack of warmth and sympathy toward others is also consistent with recent research that found individuals with higher levels of schizotypy to score higher than peers on the Self-Centered Impulsivity factor of the Psychopathic Personality Inventory-Revised [40], [41], and to self-report more physical aggression [41]. Taken together with those findings, the results from this current study have important clinical implications for treatments that attempt to improve social functioning in individuals with schizophrenia-spectrum disorders.

## Supporting Information

File S1 **Raw data SPSS file used for all analyses.**
(SAV)
